# Knowledge, Attitudes, Practices, Barriers, and Promotional Strategies Related to Clinical Data Interchange Standards Consortium Adoption Among Clinical Data Management Professionals: Semiqualitative Interview Study

**DOI:** 10.2196/84194

**Published:** 2026-06-05

**Authors:** Gaoqiang Xie, Jing He, Wenyao Ma, Phyo Kyaw Myint, Chongsheng Wu, Yangfeng Wu

**Affiliations:** 1 Department of Data Management, Clinical Research Institute, Institute of Advanced Clinical Medicine, Peking University, No.38, Xueyuan Road, Haidian District, Beijing, 100191, China, 86 01082805836, 86 010-82805836; 2 Key Laboratory of Epidemiology of Major Diseases, Peking University, Ministry of Education, , Beijing, China; 3 Aberdeen Cardiovascular & Diabetes Centre, University of Aberdeen, Aberdeen, Scotland, United Kingdom; 4 Institute of Applied Health Sciences, University of Aberdeen, Aberdeen, Scotland, United Kingdom; 5 Beijing Data Science Express Consulting Co, Ltd, Beijing, China; 6 Department of Epidemiology and Biostatistics, Peking University School of Public Health, Beijing, China

**Keywords:** Clinical Data Interchange Standards Consortium, CDISC, data management, knowledge, attitude, and practice, KAP, barriers, promotional strategies

## Abstract

**Background:**

National Medical Products Administration of China has actively encouraged organizations to adopt the Clinical Data Interchange Standards Consortium (CDISC) for clinical data submission since 2020.

**Objective:**

This study aimed to explore the knowledge, attitudes, practices, and barriers to using the CDISC among data managers and to propose strategies for promoting CDISC use in China.

**Methods:**

A total of 38 participants as leaders or experts of data management departments and teams were recruited from 38 organizations in China from April to August 2022. Data on knowledge, attitude, practice, and barriers to the CDISC, as well as strategies for its dissemination, were collected using a semiqualitative interview guide. For textual data, thematic analysis was conducted using a hybrid approach integrating constructivist and deductive logic, involving iterative coding and theme saturation checks.

**Results:**

Of the 38 participants, 36 (94.7%) mentioned that the National Medical Products Administration of China specifying CDISC standards used in submitting data is important for clinical trials, 32 (84.2%) supported this policy, and 29 (76.3%) had experience with CDISC-compliant submissions. The primary barriers to CDISC implementation included high costs, shortage of specialized expertise and training resources, inadequate localization support, inherent complexity of CDISC standards, incomplete standard specifications, lack of detailed regulatory guidelines, low overall proficiency in clinical research, heterogeneity among enterprises and institutions, and challenges related to organizational survival. Corresponding promotional strategies included cost reduction, strengthened publicity and training, adoption of the CDISC from clinical study design, development of supporting tools, integration of traditional Chinese medicine terminology into the CDISC, refinement of CDISC standards, formulation of detailed regulatory guidelines, establishment of a review system, and promotion of collaboration among enterprises and institutions.

**Conclusions:**

The CDISC has been recognized, adopted, and supported by most data management experts in China. However, key barriers to its widespread implementation continue to include cost burdens, expertise shortages, and inadequate technological, social, and policy support. Strategies to advance CDISC dissemination should prioritize improving cost-effectiveness, strengthening outreach and training, and refining regulatory frameworks. These findings provide valuable references for international regulators and sponsors in advancing the global adoption of the CDISC.

## Introduction

The Clinical Data Interchange Standards Consortium (CDISC) provides a set of core standards essential for clinical research data management [1]. Regulatory bodies including the US Food and Drug Administration and Japan’s Pharmaceuticals and Medical Devices Agency mandate the submission of clinical trial data in compliance with CDISC standards [2,3]. Following China’s accession to the International Council for Harmonization of Technical Requirements for Pharmaceuticals for Human Use (ICH) in 2017, the National Medical Products Administration (NMPA) has actively encouraged organizations to adopt the CDISC for clinical data submission since 2020 [4]. However, as a complex standardization system, CDISC implementation in China and other large countries faces multifaceted challenges, including regional adaptation, resource constraints, and institutional capacity limitations, particularly given the country’s large and heterogeneous clinical research organizations.

In clinical research, data managers are responsible for designing case report forms (CRFs), building electronic databases, managing data transmission, cleaning data, and performing CDISC-compliant data conversion. As key implementers of CDISC standards, clinical data managers play a pivotal role in the real-world application of the CDISC. In China, CDISC implementation has been reported across diverse scenarios, including CRF development and annotation [5], electronic data capture (EDC) system development and deployment [6-8], automated dataset generation [9], research in traditional Chinese medicine (TCM) [10], and studies focusing on specific disease areas [11,12]. Nevertheless, the knowledge, attitude, and practice (KAP) regarding CDISC adoption, barriers to its adoption, and strategies for its promotion remain underinvestigated in China and other countries in similar situations.

In this study, we conducted semistructured interviews with Chinese clinical data managers to characterize the KAP of CDISC adoption, identify key implementation barriers from their professional perspectives, and elicit valuable recommendations to address these challenges.

## Methods

### Ethical Considerations

This study was approved by the Biomedical Ethics Committee of Peking University (IRB00001052-21162). Verbal informed consent was acquired from all participants prior to the interview and documented via audio recording. No patient or public involvement occurred in the study design, conduct, or reporting of this research. Participants’ privacy and data confidentiality were strictly protected via deidentification of personal information. All research data were properly preserved for academic use only. Every participant received reasonable compensation in accordance with ethical requirements.

### Design

A qualitative approach featuring semistructured cross-sectional interviews was used in this study. The study protocol and semistructured guide were jointly developed by a working group consisting of GX, JH, YW, CW, and WM.

### Participants

A qualitative approach using maximum variation purposive sampling was used to recruit experts in clinical data management. Initially, 133 professionals from 103 organizations, including 47 (45.6%) pharmaceutical companies, 47 (45.6%) contract research organizations (CROs), and 9 (8.7%) academic institutions, were invited via 3 main channels: meeting attendance lists from the Clinical Research Institute of Peking University (n=70, 52.6%), membership directories of the Chinese Society of Clinical Data Management (n=29, 21.8%), and the official website of the Clinical Research Institute of Peking University together with its official WeChat Moments (n=4, 3%). Overall, 47 professionals from 47 independent organizations (n=22, 46.8% pharmaceutical companies, n=16, 34% CROs, and n=9, 19.1% academic institutions) agreed to participate in the study.

In the second stage, 44 organizations with dedicated data management teams or departments were selected, including 22 (50%) pharmaceutical companies, 14 (31.8%) CROs, and 8 (18.2%) academic institutions. Eligible experts were invited according to the following inclusion criteria: (1) holding a position as a data management leader or senior expert within their organizations and (2) providing informed consent to participate in the study. Six (13.6%) experts were unavailable or unreachable, yielding a final analytical sample of 38 (86.4%) data management experts from 38 (86.4%) distinct organizations: 13 (34.2%) pharmaceutical companies, 19 (50%) CROs, and 6 (15.8%) academic institutions.

### Data Collection

Semistructured interviews were conducted between April and August 2022. Due to geographical constraints and COVID-19 pandemic-related restrictions, the interviews were administered by trained interviewers using a mixed-mode approach (online and offline), including telephone calls, WeChat video interviews, and face-to-face meetings. The interview guide covered organizational background, participant demographics, current CDISC implementation status (including KAP), barriers encountered, and recommendations for addressing these challenges. The complete interview guide is provided in Multimedia Appendix 1.

Experts’ knowledge of the CDISC was primarily assessed through the following question: “Do you think it is important that the National Medical Products Administration of China (NMPA) specifying CDISC standards used in submitting data is important for clinical trials?” Their attitudes toward the CDISC were evaluated using the question: “Do you support the National Medical Products Administration of China (NMPA) specifying CDISC standards used in submitting data of clinical trials?” Meanwhile, experts’ practice of the CDISC was measured via the question: “Does your institute/company use CDISC standards in submission of clinical trial data?”

Prior to each interview, participants were informed that their responses would be kept confidential and anonymized. All interviews were conducted in private, quiet environments to ensure participants could communicate openly and comfortably, free from external distractions.

### Audio Data Coding

Audio files were professionally transcribed into text using the online platform “iFLYREC.” The resulting text files were systematically coded using NVivo software (Lumivero). A trained interviewer generated initial codes and deductively developed a coding framework, documenting potential connections between codes that could inform subsequent theme development.

The development of the coding framework and subsequent data interpretation were guided by the Consolidated Framework for Implementation Research, which provided a clear theoretical foundation. Three core domains were used to structure the coding scheme: (1) KAP toward CDISC use, (2) barriers to CDISC adoption, and (3) recommendations for CDISC promotion. This structure ensured a systematic and consistent approach to identifying KAP, implementation barriers, and promotion recommendations. This theoretically grounded coding strategy enhanced the rigor and transparency of the analysis, enabling the identification and interpretation of key themes within a well-established implementation science framework.

To ensure accuracy in the audio-to-text conversion and coding processes, the entire workflow was conducted by a single coder (JH) and independently reviewed and validated by a panel of 6 senior data managers. Any discrepancies were communicated to the coder (JH), followed by collaborative discussion to reach a consensus.

### Determination of Sample Size

The sample size was determined using the data saturation method, a widely adopted approach in qualitative research to ensure the comprehensiveness of collected data [11]. Recruitment was terminated once theoretical saturation was achieved [13], which referred to the point at which no new information or themes emerged from additional interviews. Data saturation was formally assessed after each of the 3 interview batches. In the first batch, 23 experts were interviewed, with only minimal new information emerging from the final 2 participants. The second batch included 11 interviews, where no new insights were generated from the last 2 experts. To further validate the achievement of saturation, a third batch of 4 interviews was conducted, which confirmed that no additional new information could be obtained. Ultimately, 38 experts completed the interviews, forming a comprehensive sample that met the criteria of theoretical saturation and ensured the adequacy of data for subsequent thematic analysis.

### Statistical Analyses

Qualitative data were analyzed using the 6-step thematic analysis method [12], which is rooted in constructivist and deductive approaches. This study adopted a constructivist paradigm, which posits that social reality is subjectively constructed via individual experiences and contextual interactions rather than being fixed or universal. This paradigm was selected to capture participants’ context-specific perspectives, a key element in addressing the research questions. In the process of analysis and interpretation, constructivism emphasized the researcher’s role in meaning-making, prioritized emergent themes over broad generalizations, and grounded findings in participants’ experiences; thus, the findings represented coconstructed, context-embedded meanings rather than absolute truths. Rejecting predetermined frameworks and causal inference, this approach centered participants’ narratives to unpack subjective factors, framing results as context-bound insights. Reflexivity, defined as reflecting on biases to preserve participants’ voices and ensure holistic data interpretation, was integrated throughout the process to guarantee the authenticity and depth of the findings, as well as alignment with the study’s purpose.

Quantitative categorical variables (eg, CDISC KAP) were described using percentages, while continuous variables were reported as mean, SD, minimum, and maximum values. This study adhered to the Consolidated Criteria for Reporting Qualitative Research (COREQ) checklist [13].

## Results

### Characteristics of Participants

A total of 38 data management experts from 38 distinct organizations participated in the study. Demographic characteristics of the experts and their affiliated companies and institutes are presented in Table 1. The experts had a mean age of 39.9 (SD 6.3) years, with 19 (50%) being male participants, 21 (55.3%) employed by international companies, 38 (100%) holding at least a bachelor’s degree, and 36 (94.7%) serving in data management leadership or higher positions within their organizations.

**Table 1.** Characteristics of individuals and companies and institutes (N=38).

Characteristics	Individuals
Characteristics of individuals	
Men, n (%)	19 (50)
Age (y), mean (SD)^a^	39.9 (6.3)
Education, n (%)	
Bachelor’s degree	10 (26.3)
Master’s degree	22 (57.9)
Doctoral degree	6 (15.8)
Position of the interviewees, n (%)	
Leader of the data management department^b^	32 (84.2)
Leader of the company or institution	4 (10.5)
Senior data manager	2 (5.3)
Type of institute or company, n (%)	
Contract research organizations	19 (50)
Pharmaceutical companies	13 (34.2)
Academic institutes	6 (15.8)
Length since establishment (y), mean (SD)	17.7 (16.2)
Characteristics of the institutes and companies, n (%)	
Staff for data management	
<20	15 (39.5)
20‐49	11 (28.9)
50‐99	4 (10.5)
>100	8 (21.1)
International company or institution	
Yes	17 (44.7)
No	21 (55.3)
Countries from which institutes originated	
Australia	2 (5.3)
Germany	1 (2.6)
United States	4 (10.5)
Switzerland	1 (2.6)
China	30 (78.9)
Provinces of China^c^	
Beijing	18 (47.4)
Guangdong	1 (2.6)
Hebei	1 (2.6)
Jiangsu	3 (7.9)
Shandong	2 (5.3)
Shanghai	11 (28.9)
Tibet	1 (2.6)
Zhejiang	1 (2.6)

a Two participants refused to answer.

b Defined as being responsible for the business of data management.

c For international institutes, province of China indicates the location of the office in China.

### KAP Toward Using the CDISC

**Figure 1.**
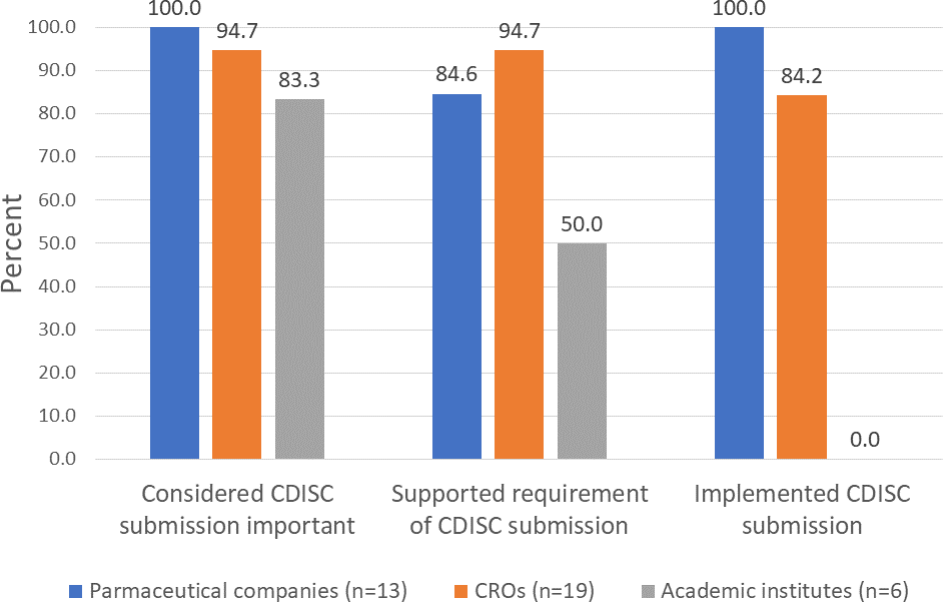
Characteristics of knowledge, attitude, and practice of the Clinical Data Interchange Standards Consortium (CDISC) in different types of companies and institutes. CRO: contract research organization.

Among the 38 experts, 36 (94.7%) mentioned that NMPA specifying CDISC standards used in submitting data is important for clinical trials, 32 (84.2%) supported this policy, and 29 (76.3%) had prior experience using the CDISC for data submission (Table 2). KAP rates varied by organizational type (Figure 1): 100% (13/13) of pharmaceutical company experts considered CDISC submission important, of whom 84.6% (11/13) supported the requirement and 100% (13/13) had implemented it; among CRO experts, 94.7% (18/19) supported the requirement and 84.2% (16/19) had implemented the CDISC; among academic experts, 83.3% (5/6) considered CDISC submission important, 50% (3/6) supported the requirement, and 0% (0/6) had implementation experience.

**Table 2.** Characteristics of knowledge, attitude, and practice in using the Clinical Data Interchange Standards Consortium (CDISC; N= 38).

Items	Values, n (%)
Considered CDISC standards being important	36 (94.7)
Supported the use of CDISC standards	32 (84.2)
Had used CDISC standards	29 (76.3)
Clinical Data Acquisition Standards Harmonization	29 (76.3)
Study Data Tabulation Model	29 (76.3)
Analysis Data Model	29 (76.3)
Define-XML	23 (60.5)
Therapeutic Areas	20 (52.6)
Controlled Terminology	20 (52.6)
Operational Data Model	2 (5.3)
Laboratory Data Model	2 (5.3)
Areas of application	
Using the CDISC in drug clinical trials	29 (76.3)
Using the CDISC in medical device clinical trials	9 (23.7)
Using the CDISC in research projects	2 (5.3)
Methods of generation	
Standard SAS program	26 (68.4)
SAS macros	22 (57.9)
Developed software or systems	7 (18.4)
Purchased software or systems	5 (13.2)
Outsourcing	2 (5.3)
R software	1 (2.6)
Measures of quality control	
Double-person programming	18 (47.4)
One person programming and another checking	4 (10.5)
System and person generation	3 (7.9)
Other methods	4 (10.5)
Number of projects using the CDISC	
0	9 (23.7)
0‐4	2 (5.3)
5‐9	2 (5.3)
10‐14	2 (5.3)
≥15	23 (60.5)

### Barriers to the CDISC, Their Mechanisms, and Proposed Strategies

**Figure 2.**
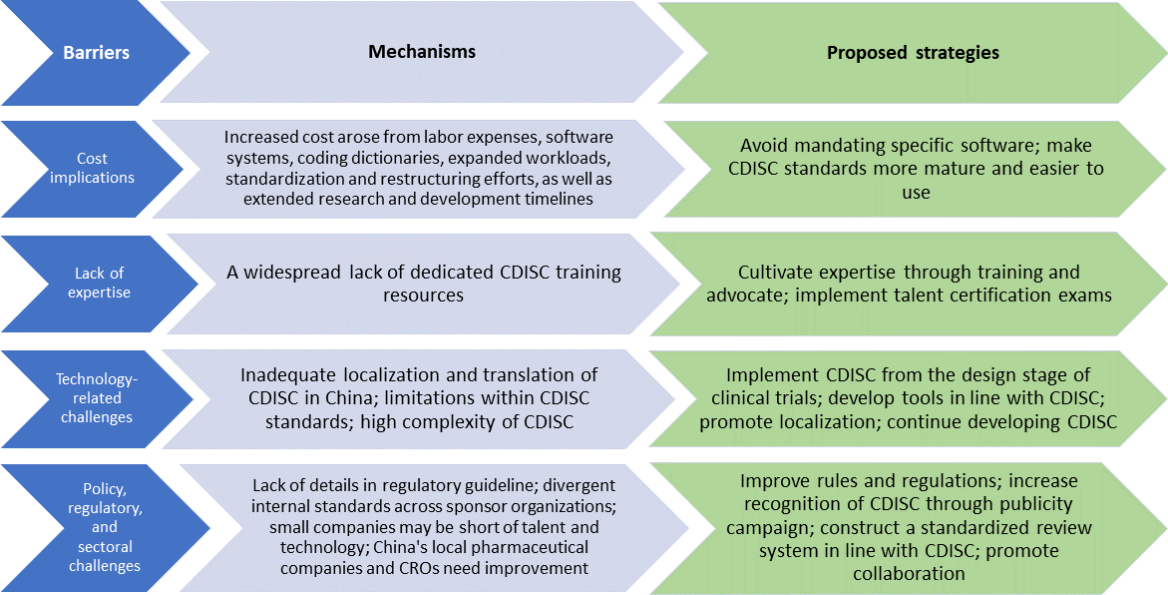
Conceptual figure on barriers to the Clinical Data Interchange Standards Consortium (CDISC), their mechanisms, and proposed strategies.

We summarized the CDISC implementation barriers, their underlying mechanisms, and proposed improvement strategies into a conceptual figure (Figure 2), and described the details in Tables3  4 and MultimediaAppendices 2  3.

**Table 3.** Barriers of using the Clinical Data Interchange Standards Consortium (CDISC) standards in the clinical data management industry.

Name of codes	Total (N=38), n (%)	Pharmaceutical companies (n=13), n (%)	Contract research organizations (n=19), n (%)	Academic institutes (n=6), n (%)
Cost factors	31 (81.6)	12 (92.3)	14 (73.7)	5 (83.3)
Expertise factors	21 (55.3)	6 (46.2)	11 (57.9)	4 (66.7)
Lack of CDISC standards expertise	21 (55.3)	6 (46.2)	11 (57.9)	4 (66.7)
Lack of training resources	2 (5.3)	0 (0)	2 (10.5)	0 (0)
Technology factors	19 (50)	3 (23.1)	10 (52.6)	6 (100)
Lack of localization	15 (39.5)	2 (15.4)	9 (47.4)	4 (66.7)
Imperfection of CDISC standards	6 (15.8)	0 (0)	5 (26.3)	1 (16.7)
Policy, regulatory, and sectoral challenges	17 (44.7)	7 (53.8)	7 (36.8)	3 (50)
Lack of detailed guidance regarding CDISC use	10 (26.3)	3 (23.1)	5 (26.3)	2 (33.3)

**Table 4.** Promotional strategies on Clinical Data Interchange Standards Consortium (CDISC) application in the clinical data management industry.

Name of codes	Total (N=38), n (%)	Pharmaceutical companies (n=13), n (%)	Contract research organizations (n=19), n (%)	Academic institutes (n=6), n (%)
Cost facet	7 (18.4)	2 (15.4)	3 (15.8)	2 (33.3)
Reducing cost as CDISC implementation matures	6 (16)	2 (15.4)	3 (15.8)	1 (16.7)
Not specifying the software, such as SAS programming software	1 (3)	0 (0)	0 (0)	1 (16.7)
Expertise facet	29 (76.3)	11 (84.6)	13 (68.4)	5 (83.3)
Cultivating expertise through advocating and training	29 (76.3)	11 (84.6)	13 (68.4)	5 (83.3)
Managing expertise through the certification qualification examination	1 (2.6)	0 (0)	0 (0)	1 (16.7)
Technology facets	29 (76.3)	7 (53.8)	16 (84.2)	6 (100)
Implementing CDISC standards from the beginning of clinical trial	19 (50)	4 (30.8)	12 (63.2)	3 (50)
Developing tools to support data processing from collection to archiving	18 (47.4)	4 (30.8)	11 (57.9)	3 (50)
Localization of CDISC standards	11 (28.9)	3 (23.1)	5 (26.3)	3 (50)
Continuing development and improvement of CDISC standards	5 (13.2)	0 (0)	5 (26.3)	0 (0)
Policy, regulatory, and sectoral challenges	33 (86.8)	12 (92.3)	16 (84.2)	5 (83.3)
Improving rules and guidelines	22 (57.9)	8 (61.5)	9 (47.4)	5 (83.3)
Increasing awareness of the CDISC through outreach and publicity	17 (44.7)	5 (38.5)	9 (47.4)	3 (50)
Constructing a standardized review system	11 (28.9)	1 (7.7)	7 (36.8)	3 (50)
Promoting collaboration among companies and institutions	6 (15.8)	5 (38.5)	1 (5.3)	0 (0)

### Barriers to CDISC Adoption

#### Cost Implications

The most frequently cited barrier was cost implications, reported by 81.6% (31/38) experts (Table 3). Increased cost arose from labor expenses, software systems, coding dictionaries, expanded workloads, standardization and restructuring efforts, as well as extended research and development timelines. Additional details can be found in Multimedia Appendix 2.

For sponsors, CDISC-compliant data submission increases programming workloads.

[Participant ID 05, CRO expert]

For companies that have already established their own standards, aligning data submissions with CDISC can be highly time-consuming due to the need to adopt an entire new framework.

[Participant ID 13, CRO expert]

Adopting CDISC-aligned data submission entails additional cost related to terminology dictionary copyright, the development of new standard operating system and the recruitment of highly specialized expertise.

[Participant ID 18, pharmaceutical industry expert]

#### Lack of Expertise

The second most frequently reported barrier was insufficient expertise, mentioned by 55.3% (21/38) of experts (Table 3). Respondents emphasized that the required skill set includes familiarity with CDISC standards, proficiency in SAS (SAS Institute) programming, knowledge of clinical operations, cross-disciplinary medical and statistical expertise, and strong communication skills. A widespread lack of dedicated CDISC training resources was identified as a key contributor to this expertise gap. Additional details can be found in Multimedia Appendix 2.

#### Technology-Related Challenges

The third most frequently reported barrier was technological support shortages, cited by 50% (19/38) of participants (Table 3). Three subthemes emerged regarding challenges associated with CDISC technology and standards. Additional details can be found in Multimedia Appendix 2.

##### Inadequate Localization of CDISC in China

Chinese translations of CDISC standards are primarily by nongovernmental organizations, and dissemination had been relatively slow.

The corresponding Chinese version lagged behind the English version.

[Participant ID 01, academic expert]

The current translations appear mechanical and lack fluency.

[Participant ID 07, pharmaceutical industry expert]

Participants also reported technical inconsistencies between CDISC standards and Chinese medical standards.

Currently, most hospital information system (HIS) in China adopt *[Chinese*] national standards that differ from CDISC requirement.

[Participant ID 30, CRO expert]

There exist terminologies specific to China which were not included in CDISC.

[Participant ID 08, pharmaceutical industry expert]

##### Limitations Within CDISC Standards

First, CDISC standards struggle to adapt rapidly to diverse clinical trial designs.

The number of variables and attributes in CDISC standards is insufficient to support comprehensive clinical studies.

[Participant ID 33, CRO expert]

Second, the development of the CDISC was perceived as deviating from formal standardization protocols.

From a standardization perspective, CDISC has not been in accordance with established standard-setting principles.

[Participant ID 36, CRO expert]

Third, inconsistencies were observed between implementation guidelines and the underlying standards.

Implementation guidelines frequently do not align with the actual standards.

[Participant ID 02, CRO expert]

##### High Complexity of the CDISC

Participants reported practical difficulties in working with CDISC datasets.

For researchers without formal CDISC training, directly using CDISC-compliant data is inherently challenging.

[Participant ID 21, academic expert]

Second, annotation files were difficult to generate, and CDISC standards were extensive and continually updated.

I find CDISC standards to be highly complex.

[Participant ID 35, pharmaceutical industry expert]

### Policy, Regulatory, and Sectoral Challenges

Of 38 experts, 17 (44.7%) identified barriers related to policy, regulatory, and sectoral factors (Table 3). Additional details can be found in Multimedia Appendix 2.

Regulatory guidelines governing CDISC application were described as lacking actionable details.

Currently, there is a lack of targeted laws and regulations for CDISC implementation, as well as a shortage of practical, on-the-ground implementation guidance.

[Participant ID 01, academic expert]

Data submission requirements from regulatory authorities remain recommendatory rather than mandatory. Regulatory agencies also require an adaptation period to identify the optimal implementation approach.

[Participant ID 06, pharmaceutical industry expert]

Divergent internal standards across sponsor organizations hinder unified adoption of the CDISC.

Pharmaceutical companies and CROs vary significantly in scale, and adopt inconsistent internal standards, making uniform CDISC compliance extremely challenging.

[Participant ID 13, CRO expert]

Notable differences also exist in data submission expectations between sponsor-led and investigator-initiated trials.

Paradoxically, studies exempt from formal data submission, mostly investigator-initiated research, present greater standardization challenges due to the absence of mandatory compliance rules.

[Participant ID 16, CRO expert]

Gaps in talent, financial strength, and management experience also exist between large and small organizations. Smaller companies may face industry attrition risks if they cannot recruit personnel or establish technical systems capable of supporting the CDISC.

If CDISC-based data submission becomes mandatory, teams and companies capable of meeting these standards will be immediately distinguished. Those lacking adequate capacity may face elimination, along with under-skilled professionals within the sector.

[Participant ID 14, CRO expert]

Data management capabilities among domestic Chinese pharmaceutical companies and CROs require further improvement.

To my knowledge, some local pharmaceutical companies have not yet established or full refined data management-related standard operating procedures (SOPs). Achieving standardized data submission will require substantial efforts to improve both data quality and overall trial quality.

[Participant ID 26, pharmaceutical industry expert]

### Promotional Strategies for the CDISC

Table 4 summarizes key promotional strategies from participants to advance CDISC adoption and address identified barriers. Additional details can be found in Multimedia Appendix 3.

First, of 38 experts, 33 (86.8%) proposed one or more suggestions in the policy, regulatory, and sectoral challenges facet: (1) expedite the development of detailed rules and regulations to support CDISC implementation, (2) enhance industry awareness of the CDISC through targeted outreach and education, (3) establish a CDISC-aligned standardized review system, and (4) strengthen cross-sector collaboration.

There is an antagonistic relationship between the industry and regulation, and regulators should conduct more outreach to facilitate industry acceptance of this standard.

[Participant ID 05, CRO expert]

The NMPA *[National Medical Products Administration*] should develop dedicated software to efficiently verify CDISC compliance of submitted data.

[Participant ID 10, CRO expert]

Currently, SDTM generation and mapping work is entirely outsourced to third-party vendors.

[Participant ID 37, pharmaceutical industry expert]

Second, out of 38 experts, 29 (76.3%) proposed two talent-related strategies: (1) expand CDISC training and advocacy to build specialized expertise, with an emphasis on affordable, high-quality programs and (2) implement professional certification programs to standardize workforce competencies.

We need more training resources and additional accredited training institutions as soon as possible.

[Participant ID 14, CRO expert]

A formal CDISC certification examination should be established.

[Participant ID 15, academic expert]

Third, of 38 experts, 29 (76.3%) proposed four technology-focused solutions: (1) integrate CDISC requirements from the clinical trial design phase, (2) develop tools in line with the CDISC, (3) advance CDISC localization, and (4) continue developing the CDISC. Establishing a standardized CRF library and corresponding standard operating procedures can improve efficiency and reduce barriers to CDISC-compliant data generation.

If we want to make the standardization process smoother, solving it from the source may be optimal.

[Participant ID 06, pharmaceutical industry expert]

We need some resources such as CDISC-aligned software systems.

[Participant ID 09, CRO expert]

CDISC standards should be adapted to better suit for China.

[Participant ID 10, academic expert]

CDISC should be expanded to cover additional therapeutic areas and indications.

[Participant ID 33, CRO expert]

Fourth, of 38 experts, 7 (18.4%) proposed cost-related adjustments: (1) avoid mandating specific software and (2) leverage long-term efficiency gains to offset initial investments.

Data converted using alternative programming software should also be acceptable for regulatory submission.

[Participant ID 15, academic expert]

As CDISC implementation matures, overall research and development costs may decrease.

If regulatory review time can be shortened, overall R&D costs will be declined.

[Participant ID 34, academic expert]

From a CRO perspective, CDISC alignment improves data management standardization, efficient, and data quality. While CDISC implementation may be troublesome initially, substantial long-term benefits can be realized.

[Participant ID 24, CRO expert]

Among the abovementioned suggestions, expertise development and strengthening policy and environmental support were most frequently emphasized by pharmaceutical industry experts. Cost control and technical problem-solving were prioritized by academic respondents.

## Discussion

### Principal Findings

In this qualitative study involving 38 Chinese clinical data management experts, 36 (94.7%) regarded the specification of CDISC standards for regulatory data submission by the NMPA as important for clinical trials, 32 (84.2%) supported CDISC adoption for data submission, and 29 (76.3%) reported prior experience with CDISC-compliant submissions. Key barriers to CDISC implementation and dissemination included increased implementation costs, shortages of specialized expertise, and insufficient technical, socioenvironmental, and policy supports. Corresponding strategies should focus on mitigating cost impacts, improving CDISC implementation efficiency, strengthening publicity and training, and improving regulatory frameworks. To our knowledge, this is the first study exploring KAP, barriers, and promotional strategies for CDISC implementation among Chinese data management professionals. It provides valuable insights for advancing CDISC adoption within the clinical data management sector in China and other countries with similar situations.

This study yields several important findings. First, it represents the first systematic characterization of perspectives among Chinese data management experts toward CDISC promotion and application, with most expressing support. As data management experts and their teams represent the primary end users of CDISC standards, these findings indicate favorable societal and professional support for CDISC implementation in China. Second, through qualitative expert inquiry, we identified core challenges hindering CDISC uptake, providing clear targets for finding measures to overcome these difficulties. Third, we derived actionable policy and operational strategies for promoting CDISC dissemination, which can inform future regulatory and industry guidance. Fourth, while barriers and promotional strategies were broadly consistent across the pharmaceutical industry, CRO, and academic experts, relative emphasis differed: pharmaceutical industry experts prioritized cost, policy, and environmental factors, whereas academic respondents focused more on expertise and technical challenges. These differences likely reflect industry reliance on commercial drug development revenue compared with the diversified funding sources of academic institutions, including government grants and industry sponsorship. We believe these findings provide useful promotion strategies to support CDISC implementation in China and other countries in similar situations.

This study carries important practical implications. First, it can encourage technical advances in automated CDISC data conversion, including low-cost or open-source CDISC-compliant EDC systems [14], such as OpenEDC; semiautomated legacy data conversion (clinical trial legacy data into CDISC Study Data Tabulation Model [SDTM] standards format) using supervised machine learning; and standards-based electronic regulatory submission frameworks [15,16]. Second, it supports timely and high-quality translation and localization of the CDISC, including alignment with Chinese electronic medical records and TCM standards. Although Chinese versions of SDTM, Analysis Data Model, and Controlled Terminology are available via the CDISC website, Chinese terminology coverage remains less than 1% of that of the English version. Chinese TCM experts are actively exploring CDISC application in this domain [17-21]. Third, the findings can inform the refinement of CDISC-related regulations. The United States and Japan have established comprehensive regulatory frameworks to enforce the CDISC [2,3], and the European Union has formally announced plans to adopt the CDISC for regulatory review [22]. While Chinese regulators have recommended CDISC-aligned submission, automation in clinical data management remains limited due to the lack of detailed submission guidelines. As China joins ICH and aligns drug research and development with international markets, CDISC implementation is increasingly inevitable. On the basis of these findings, we recommend that Chinese regulators adopt a phased rollout (eg, SDTM first, followed by Analysis Data Model; voluntary adoption preceding mandatory compliance), establish pilot submission programs, publish contextually appropriate technical conformance guides, and develop regulator-sponsored validation tools.

The sample was determined using the principle of theoretical saturation, whereby no new themes or conceptual insights emerged with additional interviews. Interviews were conducted in 3 batches (23, 11, and 4 participants, respectively) using a data-driven adaptive sampling strategy to evaluate saturation progressively rather than using a fixed recruitment plan. Saturation was confirmed after the third batch, with no new themes identified, supporting the adequacy of the final sample size and ensuring rigor in the qualitative analysis. Determining the sample size based on theoretical saturation not only meets research needs but also avoids unnecessarily expanding the sample size and increasing the research burden.

### Strengths and Limitations

The study has several methodological strengths. First, the study was designed scientifically and rigorously by a multidisciplinary team with expertise in the CDISC, data management, and clinical research methodology. Second, sample size was determined using the information saturation method, with data reaching thematic saturation. Third, strict quality control procedures were implemented to ensure research credibility. Fourth, the study was carried out under the supervision of an ethics committee to ensure the privacy and rights of participants. Finally, reporting followed the COREQ checklist for transparent qualitative research reporting.

Several limitations should be acknowledged. First, representativeness was constrained by the unequal distribution of organizational types in the final sample (n=38): 19 (50%) CROs, 13 (34.2%) pharmaceutical companies, and only 6 (15.8%) academic institutions. Because none of the academic experts reported CDISC submission experience, the relatively small academic subsample may have led to overestimation of overall CDISC implementation rates. Similarly, because academic experts prioritized technical and expertise-related barriers over cost, the underrepresentation of academic experts may have resulted in underestimation of technical and expertise barriers and overestimation of cost-related challenges. Second, participants were predominantly senior data management professionals, and many from large or international organizations, chosen for their in-depth understanding of CDISC barriers and strategic recommendations. However, this participant composition limited data collection on KAP regarding the CDISC among junior data managers from smaller domestic organizations, who may have lower CDISC literacy. This may have led to overestimation of sector-wide CDISC-related KAP. Nevertheless, maximum variation purposive sampling was applied, and data reached theoretical saturation; thus, the findings remain highly informative, particularly regarding CDISC implementation barriers and promotional strategies.

### Conclusions

The CDISC is already in use among many participating experts and their organizations, but the depth of implementation remains limited. Challenges to CDISC adoption in China span 4 domains: financial constraints, workforce skill gaps, inadequate technical support, and underdeveloped policy and regulatory environments. Strategies to promote the CDISC in China include mitigating cost burdens, expanding professional training, integrating the CDISC from the study design stage, developing automated software tools, improving localization and translation, supporting continued evolution of CDISC standards, refining regulations and implementation guidelines, establishing CDISC-aligned regulatory review systems, and strengthening cross-organizational collaboration.

## Supplementary material

10.2196/84194 Multimedia Appendix 1 Interview guide on knowledge, attitudes, and practices, barriers and promotion strategies of the Clinical Data Interchange Standards Consortium among clinical data management experts in China.

10.2196/84194 Multimedia Appendix 2 Details of barriers of using Clinical Data Interchange Standards Consortium standards in clinical data management sector.

10.2196/84194 Multimedia Appendix 3 Details of strategies to promote Clinical Data Interchange Standards Consortium application in clinical data management industry.

## Abbreviations

CDISC: Clinical Data Interchange Standards Consortium
COREQ: Consolidated Criteria for Reporting Qualitative Research
CRF: case report form
CRO: contract research organization
EDC: electronic data capture
ICH: International Council for Harmonization of Technical Requirements for Pharmaceuticals for Human Use
NMPA: National Medical Products Administration of China
SDTM: Study Data Tabulation Model

## References

[1] Founder and president emeritus. Clinical Data Interchange Standards Consortium.

[2] Study data technical conformance guide. U.S. Department of Health and Human Services.

[3] (2022). Technical conformance guide on electronic study data submissions. Pharmaceuticals and Medical Devices Agency.

[4] (2020). Guiding principles for submission of drug clinical trial data. National Medical Products Administration.

[5] Wang HW, Deng YZ, Liu D (2015). Process and method for CDISC-based CRF annotation [Article in Chinese]. Yao Xue Xue Bao.

[6] Xiao Q, Wan X, Hui C, Jiao T, Ping N, Linyong X (2016). Construction of standardized data capture system and its application in quality control of clinical trial data [Article in Chinese]. Chin J Clin Pharmacol Ther.

[7] Geng L, Xiaoyan L, Zehuai W (2017). Application of Clinical Data Interchange Standards Consortium (CDISC) standards to electronic data capture system [Article in Chinese]. World Sci Technol Tradit Chin Med Mater Medica.

[8] Ye C Application of international clinical data exchange standards in vaccine clinical trials under electronic data collection system [Article in Chinese]. https://d.wanfangdata.com.cn/conference/CiFDb25mZXJlbmNlTmV3U29scjlTMjAyNjA1MjgxMDAxNDESBzk4ODk1NzgaCG9ldnN2MTFm.

[9] Chongxu W (2018). Electronic data management and CDISC data automation in clinical trials [Master's thesis in Chinese]. https://d.wanfangdata.com.cn/thesis/Ch1UaGVzaXNOZXdTb2xyOVMyMDI2MDUxMjA3Mjk0NxIJRDAxNjU5OTIzGgg2ZzRxanU5ZA%3D%3D.

[10] Fang L, Qingna L, Yang Z, Rui G (2016). A preliminary discussion of application of CDISC standards in clinical research data management of new traditional Chinese medicine [Article in Chinese]. Chin J Inf TCM.

[11] Glaser BG, Strauss AL, Strutzel E (1968). The discovery of grounded theory; strategies for qualitative research. Nurs Res.

[12] Nowell LS, Norris JM, White DE, Moules NJ (2017). Thematic analysis: striving to meet the trustworthiness criteria. Int J Qual Methods.

[13] Tong A, Sainsbury P, Craig J (2007). Consolidated criteria for reporting qualitative research (COREQ): a 32-item checklist for interviews and focus groups. Int J Qual Health Care.

[14] Greulich L, Hegselmann S, Dugas M (2021). An open-source, standard-compliant, and mobile electronic data capture system for medical research (OpenEDC): design and evaluation study. JMIR Med Inform.

[15] Oda T, Chiu SW, Yamaguchi T (2021). Semi-automated conversion of clinical trial legacy data into CDISC SDTM standards format using supervised machine learning. Methods Inf Med.

[16] Lin CH, Chou HI, Yang UC (2018). A standard-driven approach for electronic submission to pharmaceutical regulatory authorities. J Biomed Inform.

[17] Fang L, Rui G, Xudong T, Rui L (2011). CDISC as a data management standard in clinical research and its application prospects [Article in Chinese]. Chin J New Drugs.

[18] Jinghua L, Meng C, Haiyan L, Jing L, Hongjie G, Qi Y (2015). Discussion on CDISC standard and development of TCM clinical trial data interchange standard [Article in Chinese]. Chin J Libr Inf.

[19] Yanke AI, Liyun HE, Tiancai WE, Dongning WU, Baoyan LI (2015). Approach to CDISC SDTM implementation for clinical trials data submission [Article in Chinese]. World Sci Technol Mod Tradit Chin Med.

[20] Yanlan L (2013). Discussion on the establishment of standard data system for clinical research of Chinese medicine under the framework of CDISC [Master’s thesis in Chinese]. https://d.wanfangdata.com.cn/thesis/Ch1UaGVzaXNOZXdTb2xyOVMyMDI2MDUxMjA3Mjk0NxIIWTIyOTkxMzMaCHZ5NXpxcWJy.

[21] Li G, Li X, Wen Z (2014). Application of clinical data interchange standards consortium standards in the design of case report forms for clinical research in traditional Chinese medicine [Article in Chinese]. J Guangzhou Univ Tradit.

[22] (2013). Publication and access to clinical-trial data. European Medicines Agency.

