# Supplementary material for: Knowledge, Attitudes, Practices, Barriers, and Promotional Strategies Related to Clinical Data Interchange Standards Consortium Adoption Among Clinical Data Management Professionals: Semiqualitative Interview Study
Source: JMIR Med Inform. 2026 Jun 5;14:e84194. doi: 10.2196/84194 (PMC13240979; doi:10.2196/84194)
Supplement: Multimedia Appendix 1 [file medinform-v14-e84194-s001.pdf]

Multimedia appendix 1. Interview Guide on Knowledge, Attitudes, Practice, Barriers and Promotion Strategies of CDISC Among Clinical Data Management Experts in China

Individual Information Form

1. The Name of the Subject: ______________________
2. The ID Number of the Subject: ________________________
3. The Abbreviation of the Subject's Name: ________________________
4. E-mail: ______________________
5. Telephone: _________________________
6. The Mailing Address: ____
7. WeChat ID:_________________________________

Questionnaire on Knowledge, Attitudes, Practice, Barriers and Promotion Strategies of CDISC Among Clinical Data Management Experts in China

Dear Sir/Madam：

Hello！ Thank you for completing this interview. The purpose of this questionnaire is to understand the current status, obstacles and the prospect of the application of CDISC standards in China and the questionnaire will be only used for academic research, all contents are strictly confidential. Please answer and express your views seriously according to the actual situation, thank you for your support and help!

The Date of Survey： □□□□ Year □□ Month □□ Day

1. The ID Number of the Subject： ________________________
2. The Abbreviation of the Subject's Name： ________________________
3. The Name of the Subject's Institute/Company： ______________________
4. Is there data management department in your institute/company？

   ○Yes(If you choose Yes, Please answer question:5-26)

   ○No
5. Is your institute/company international?

   ○Yes

   ○No
6. What is the attribute of your institute/company?

   ○CRO

   ○Pharmaceutical/Medical Device Company

   ○Academic Institution

   ○Others:______________
7. What is the year of establishment of your institute/company： □□□□Year
8. What is the scale of your data management department：

   ○<20 Persons

   ○20-49 Persons

   ○50-99 Persons

   ○100 Persons and Above
9. What year were you born in： □□□□ Year
10. What is your degree： ○Bachelor ○Master ○Doctor ○Others___________
11. What is your sex： ○Male ○Female

12. In your opinion, is it important to mandate the adoption of CDISC standards for data submission in clinical trials intended for marketing authorization applications in China?

○Yes, please tell me the most important values that you think(qualitative investigation)

○No, why?

13. Do you support the mandatory adoption of CDISC standards for data submission to the medical products regulatory agency in clinical trials for marketing authorization?

○Yes, why?

○No, why?

14. Has your institution ever adopted CDISC standards in clinical trial data submission?

○Yes, why? Answer questions:15-19

○No, why? Answer questions:20-21

15. At present, which CDISC standards does your institute/company use ？

☐SEND

☐CDASH

☐SDTM

☐ADaM

☐TA

☐CT

☐Define-XML

☐Others, please describe in detail________________________。

16. How many studies does your institute/company use CDISC standards.

○ < 5

○5-9

○10-14

○≥15

17. Which areas does your institute/company use the CDISC standards in：

☐Scientific Research Projects

☐Drug Clinical studies

☐Medical Device Clinical studies

18. At present, how does your institute/company convert the data into CDISC formats(SDTM/ADaM)：

☐SAS program

☐SAS macro program

☐Data conversion software/system independently developed by your institute/company

☐Data conversion software/system by purchasing

☐Others:_______________

19. What are the methods to ensure the data complies with CDISC standards?

〇Two persons generate data sets according to CDISC standards and compare with each other until they are consistent;

〇One person generates data sets according to CDISC standards and another one checks and corrects

〇One person generates data sets according to CDISC standards and then compares them with data sets generated from the system until they are consistent

〇Others, please describe in detail____________________

20. Does your institute/company has the plan to submit data according to CDISC standards in the future?

〇Yes, how many years later does your institute/company plan to use to submit data according to CDISC standards in the future

〇No, why?

21. In the future, how does your institute/company will convert the data into CDISC formats , such as SDTM/ADaM:

☐SAS program

☐SAS macro program

☐Data conversion software/system independently developed by your institute/company

☐Data conversion software/system by purchasing

☐Others:______________

22. What impact on the data management industry will do you think if use of CDISC standards is mandatory?
23. What do you think about the difficulties if the use of CDISC standards is mandatory in data submissions in China?
24. What are your suggestions for solving the difficulties above?
25. What do you think about the prospect of the comprehensive promotion and application of CDISC standards in China?
26. What suggestions do you have for promoting the application of CDISC standards in China?
