# Supplementary material for: Knowledge, Attitudes, Practices, Barriers, and Promotional Strategies Related to Clinical Data Interchange Standards Consortium Adoption Among Clinical Data Management Professionals: Semiqualitative Interview Study
Source: JMIR Med Inform. 2026 Jun 5;14:e84194. doi: 10.2196/84194 (PMC13240979; doi:10.2196/84194)
Supplement: Multimedia Appendix 2 [file medinform-v14-e84194-s002.docx]

**Multimedia appendix 2.** **Details of barriers of using CDISC standards in clinical data management sector**

| The Name of Codes | Respondents/Total (%) | | | | | | | |
| --- | --- | --- | --- | --- | --- | --- | --- | --- |
|  | Total  (N=38) | Types of Companies/Institutes | | |  | Regions | | |
|  |  | Pharmaceutical Companies  (N=13) | CROs  (N=19) | Academic Institutes (N=6) |  | Beijing  (N=18) | Shanghai  (N=11) | Others  (N=9) |
| Cost factors | 31/38(81.6) | 12/13(92.3) | 14/19(73.7) | 5/6(83.3) |  | 13/18(72.2) | 9/11(81.8) | 9/9(100) |
| Expertise factors | 21/38(55.3) | 6/13(46.2) | 11/19(57.9) | 4/6(66.7) |  | 11/18(61.1) | 4/11(36.4) | 5/9(55.6) |
| Being lack of CDISC standards expertises | 21/38(55.3) | 6/13(46.2) | 11/19(57.9) | 4/6(66.7) |  | 11/18(61.1) | 4/11(36.4) | 5/9(55.6) |
| Being lack of training resources | 2/38(5.3) | 0/13(0) | 2/19(10.5) | 0/6(0) |  | 1/18(5.6)) | 1/11(9.1) | 0/9(0) |
| Technology factors | 19/38(50) | 3/13(23.1) | 10/19(52.6) | 6/6(100) |  | 11/18(61.1) | 4/11(36.4) | 4/9(44.4) |
| Being lack of localization | 15/38(39.5) | 2/13(15.4) | 9/19(47.4) | 4/6(66.7) |  | 8/18(44.4) | 3/11(27.3) | 4/9(44.4) |
| Being lack of normative in Chinese version | 5/38(13.2) | 1/13(7.7) | 3/19(15.8) | 1/6(16.7) |  | 1/18(5.6)) | 1/11(9.1) | 3/9(33.3) |
| Being slow to translate into Chinese version | 5/38(13.2) | 3/13(23.1) | 2/19(10.5) | 0/6(0) |  | 2/18(11.1) | 2/11(18.2) | 1/9(11.1) |
| The differences between the traditional Chinese medicine standards and CDISC standards | 5/38(7.9) | 0/13(0) | 2/19(10.5) | 3/6(50) |  | 3/18(16.7) | 1/11(9.1) | 1/9(11.1) |
| The differences between electronic medical records standards and CDISC standards | 3/38(7.9) | 0/13(0) | 2/19(10.5) | 1/6(16.7) |  | 2/18(11.1) | 0/11(0) | 1/9(11.1) |
| Being lack of controlled terminology specific to China | 1/38(2.6) | 1/13(7.7) | 0/19(0) | 0/6(0) |  | 0/18(0) | 0/11(0) | 1/9(11.1) |
| The imperfection of CDISC standards | 6/38(15.8) | 0/13(0) | 5/19(26.3) | 1/6(16.7) |  | 5/18(27.8) | 1/11(9.1) | 0/9(0) |
| Could not rapidly adapt to the diversity and rapid development of clinical trials | 4/38(18.4) | 0/13(0) | 3/19(15.8) | 1/6(16.7) |  | 3/18(16.7) | 1/11(9.1) | 0/9(0) |
| Being lack of normative in CDISC development | 2/38(5.3) | 0/13(0) | 2/19(10.5) | 0/6(0) |  | 2/18(11.1) | 0/11(0) | 0/9(0) |
| Being not corresponding between guidelines and its standards | 1/38(2.6) | 0/13(0) | 1/19(5.3) | 0/6(0) |  | 1/18(5.6)) | 0/11(0) | 0/9(0) |
| High complexity of CDISC standards | 6/38(15.8) | 1/13(7.7) | 3/19(15.8) | 2/6(33.3) |  | 4/18(22.2) | 1/11(9.1) | 1/9(11.1) |
| Being not easily used by data users | 3/38(7.9) | 1/13(7.7) | 1/19(5.3) | 1/6(16.7) |  | 1/18(5.6)) | 1/11(9.1) | 1/9(11.1) |
| The complexity of the annotation files | 1/38(2.6) | 0/13(0) | 1/19(5.3) | 0/6(0) |  | 0/18(0) | 1/11(9.1) | 0/9(0) |
| Being updated constantly | 1/38(2.6) | 0/13(0) | 0/19(0) | 1/6(16.7) |  | 1/18(5.6)) | 0/11(0) | 0/9(0) |
| The sheer size of the CDISC standards system | 1/38(2.6) | 0/13(0) | 0/19(0) | 1/6(16.7) |  | 1/18(5.6)) | 0/11(0) | 0/9(0) |
| Policy, regulatory, and sectoral challenges factor | 17/38(44.7) | 7/13(53.8) | 7/19(36.8) | 3/6(50) |  | 7/18(38.9) | 7/11(63.6) | 3/9(33.3) |
| Being lack of details in the requirements on using CDISC | 10/38(26.3) | 3/13(23.1) | 5/19(26.3) | 2/6(33.3) |  | 5/18(27.8) | 3/11(27.3) | 2/9(22.2) |
| Being imperfect in the review system of regulators | 6/38(15.8) | 1/13(7.7) | 3/19(15.8) | 2/6(33.3) |  | 3/18(16.7) | 2/11(18.2) | 1/9(11.1) |
| Being lack of details in rules/ regulations | 5/38(13.2) | 2/13(15.4) | 3/19(15.8) | 0/6(0) |  | 2/18(11.1) | 2/11(18.2) | 1/9(11.1) |
| Facing short-term fluctuation in the industry of clinical data management | 7/38(18.4) | 1/13(7.7) | 3/19(15.8) | 3/6(50) |  | 4/18(22.2) | 2/11(18.2) | 1/9(11.1) |
| The diversity of sponsors | 3/38(5.3) | 2/13(15.4) | 1/19(5.3) | 0/6(0) |  | 1/18(5.6)) | 1/11(9.1) | 1/9(11.1) |
| The level of clinical trials being not too high | 2/38(5.3) | 2/13(15.4) | 0/19(0) | 0/6(0) |  | 0/18(0) | 2/11(18.2) | 0/9(0) |
