# Supplementary material for: Knowledge, Attitudes, Practices, Barriers, and Promotional Strategies Related to Clinical Data Interchange Standards Consortium Adoption Among Clinical Data Management Professionals: Semiqualitative Interview Study
Source: JMIR Med Inform. 2026 Jun 5;14:e84194. doi: 10.2196/84194 (PMC13240979; doi:10.2196/84194)
Supplement: Multimedia Appendix 3 [file medinform-v14-e84194-s003.docx]

**Multimedia appendix 3.** **Details of strategies to promote CDISC application in clinical data management industry**

| The Name of Codes | Respondents/Total (%) | | | | | | | |
| --- | --- | --- | --- | --- | --- | --- | --- | --- |
|  | Total  (N=38) | Types of Companies/Institutes | | |  | Regions | | |
|  |  | Pharmaceutical Companies  (N=13) | CROs  (N=19) | Academic Institutes (N=6) |  | Beijing  (N=18) | Shanghai  (N=11) | Others  (N=9) |
| The cost facet | 7/38(7.9) | 2/13(15.4) | 3/19(15.8) | 2/6(33.3) |  | 5/18(27.8) | 1/11(9.1) | 1/9(11.1) |
| Reducing cost after maturation of application | 6/38(15.8) | 2/13(15.4) | 3/19(15.8) | 1/6(16.7) |  | 4/18(22.2) | 1/11(9.1) | 1/9(11.1) |
| Not specifying the software such as the programming software: SAS | 1/38(2.6) | 0/13(0) | 0/19(0) | 1/6(16.7) |  | 1/18(5.6) | 0/11(0) | 0/9(0) |
| The expertise facet | 29(76.3) | 11(84.6) | 13(68.4) | 5(83.3) |  | 14(77.7) | 8(72.7) | 7(77.8) |
| Cultivating expertise through advocating and training | 29(76.3) | 11(84.6) | 13(68.4) | 5(83.3) |  | 14(77.7) | 7(63.6) | 7(77.8) |
| Building a platform of training in CDISC standards | 14(36.8) | 5(38.5) | 7(36.8) | 2(33.3) |  | 5(27.8) | 6(54.5) | 3(33.3) |
| Providing diverse training contents such as CDISC standards, programming, and coding dictionaries being included | 11(28.9) | 3(23.1) | 6(31.6) | 2(33.3) |  | 4(22.2) | 3(27.3) | 2(22.2) |
| Promoting the expertise’ understanding and use of the CDISC standards through advocacy | 8(21.1) | 1(7.7) | 4(21.1) | 3(50) |  | 6(33.3) | 0(0) | 2(22.2) |
| Providing more cheaper, affordable, higher quality, extensive and official training | 7(18.4) | 2(15.4) | 4(21.1) | 1(16.7) |  | 4(22.2) | 2(18.2) | 1(11.1) |
| Managing expertise through the certification qualification examination | 1(2.6) | 0(0) | 0(0) | 1(16.7) |  | 1(5.6) | 0(0) | 0(0) |
| The technology facets | 29(76.3) | 7(53.8) | 16(84.2) | 6(100) |  | 17(94.4) | 5(45.4) | 7(77.8) |
| Implementing CDISC standards form the beginning of clinical trial | 19(50) | 4(30.8) | 12(63.2) | 3(50) |  | 12(66.7) | 3(27.3) | 4(44.4) |
| Constructing case report form(CRF) libraries | 8(21.1) | 2(15.4) | 6(31.6) | 0(0) |  | 4(22.2) | 2(18.2) | 2(22.2) |
| Constructing a standard operating system | 7(18.4) | 2(15.4) | 4(21.1) | 1(16.7) |  | 4(22.2) | 1(9.1) | 2(22.2) |
| Developing tools to help the data processing from the collection to archive | 18(47.4) | 4(30.8) | 11(57.9) | 3(50) |  | 10(55.6) | 4(36.4) | 4(44.4) |
| Localization of CDISC standards | 11/38(44.7) | 3/13(23.1) | 5/19(26.3) | 3/6(50) |  | 5/18(27.8) | 2/11(18.2) | 4/9(44.4) |
| Docking CDISC standards with Chinese medical standards. | 5/38(13.2) | 1/13(7.7) | 2/19(10.5) | 2/6(33.3) |  | 3/18(16.7) | 0/11(0) | 2/9(22.2) |
| Translating English of CDISC standards into Chinese timely and standardized. | 5/38(13.2) | 1/13(7.7) | 3/19(15.8) | 1/6(16.7) |  | 3/18(16.7) | 1/11(9.1) | 1/9(11.1) |
| Developing Controlled Terminology specific to China. | 3/38(10.5) | 1/13(7.7) | 1/19(5.3) | 1/6(16.7) |  | 1/18(5.6) | 1/11(9.1) | 1/9(11.1) |
| Continuing to develop and improve CDISC standards | 5(13.2) | 0(0) | 5(26.3) | 0(0) |  | 4(22.2) | 1(9.1) | 0(0) |
| Standardizing the construction of CDISC standards in the way of standardization | 2/38(5.3) | 0/13(0) | 2/19(10.5) | 0/6(0) |  | 2/18(11.1) | 0/11(0) | 0/9(0) |
| Expanding application scope of CDISC | 2/38(5.3) | 0/13(0) | 2/19(10.5) | 0/6(0) |  | 2/18(11.1) | 0/11(0) | 0/9(0) |
| Speeding up CDISC standards updating | 3(7.9) | 0(0) | 3(15.8) | 0(0) |  | 2(11.1) | 1(9.1) | 0(0) |
| Policy, regulatory, and sectoral challenges factors | 33/38(86.8) | 12/13(92.3) | 16/19(84.2) | 5/6(83.3) |  | 14/18(77.8) | 11/11(100) | 8/9(88.9) |
| Improving rules/ guidelines | 22/38(57.9) | 8/13(61.5) | 9/19(47.4) | 5/6(83.3) |  | 10/18(55.6) | 6/11(54.5) | 6/9(66.7) |
| Increasing the recognition to CDISC by actively publicizing | 17/38(44.7) | 5/13(38.5) | 9/19(47.4) | 3/6(50) |  | 8/18(44.4) | 4/11(36.4) | 5/9(55.6) |
| Constructing the standardized review system | 11/38(29) | 1/13(7.7) | 7/19(36.8) | 3/6(50) |  | 7/18(38.9) | 3/11(27.3) | 1/9(11.1) |
| Promoting collaborations among companies/institutes | 6/38(15.8) | 5/13(38.5) | 1/19(5.3) | 0/6(0) |  | 0/18(0) | 5/11(45.4) | 1/9(11.1) |
